# Marginal and internal fit of full ceramic crowns milled using CADCAM systems on cadaver full arch scans

**DOI:** 10.1186/s12903-020-01181-9

**Published:** 2020-07-06

**Authors:** János Vág, Zsolt Nagy, Christopher Bocklet, Tamás Kiss, Ákos Nagy, Botond Simon, Ákos Mikolicz, Walter Renne

**Affiliations:** 1 Department of Conservative Dentistry, Semmelweis University, Szentkirályi utca 47, Budapest, H-1088 Hungary; 2 College of Dental Medicine, Medical University of South Carolina, Charleston, SC USA; 3 János Szentágothai Research Centre & Department of Pharmacology and Pharmacotherapy, Medical School, University of Pecs, Ifjúság útja 20, Pécs, H-7624 Hungary; 4 Department of Dentistry, Oral and Maxillofacial Surgery, University of Pecs, Pecs, Hungary; 5 Department of Oral Rehabilitation, College of Dental Medicine, Medical University of South Carolina, Charleston, SC USA

**Keywords:** Digital impression, Intraoral scan, Accuracy, Marginal gap, Marginal fit, Chairside CADCAM, Full-ceramic crown

## Abstract

**Background:**

Chairside systems are becoming more popular for fabricating full-ceramic single restorations, but there is very little knowledge about the effect of the entire workflow process on restoration fit. Therefore, this study aimed to compare the absolute marginal discrepancy (AMD) and the full internal fit (FULL) of all-ceramic crowns made by two chairside systems, Planmeca FIT and CEREC, with detailed and standard mill settings.

**Methods:**

One upper molar was prepared for an all-ceramic crown in human cadaver maxilla. Full-arch scans were made by Emerald or Omnicam four times each. Twenty-four e.max crowns were designed and milled by the Planmill 30s or 40s or CEREC MCXL mills with either detailed or standard settings. The cadaver tooth was extracted, and each crown was fixed on it and scanned by a high-resolution microCT scanner. The AMD and FULL were measured digitally in mesio-distal and bucco-lingual 2D slices. The actual and predicted times of the milling were also registered.

**Results:**

No differences were observed between detailed or standard settings in either system. The AMD was significantly higher with CEREC (132 ± 12 μm) than with either Planmill 30s (71 ± 6.9 μm) or 40s (78 ± 7.7 μm). In standard mode, the FULL was significantly higher with CEREC (224 ± 9.6 μm) than with either Planmill 30s (169 ± 8.1 μm) or 40s (178 ± 8.5 μm). There was no difference between actual and predicted time with the two Planmeca models, but with CEREC, the actual time was significantly higher than the predicted time. The 30s had significantly higher actual and predicted times compared to all other models. Across all models, the average milling time was 7.2 min less in standard mode than in detailed mode.

**Conclusions:**

All fit parameters were in an acceptable range. No differences in fit between Planmeca models suggest no effect of spindle number on accuracy. The detailed setting has no improvement in the marginal or internal fit of the restoration, yet it increases milling time.

## Background

In modern dentistry, the 3D scanning and modeling capabilities allow design work to be done digitally chairside instead of in a traditional laboratory setting. The combination of digital design and machine manufacturing techniques is termed computer-aided design/computer-aided manufacturing (CADCAM). Current literature has extensively investigated the fit of conventionally fabricated fixed restorations, both at the restoration margin and internal surface [1–5]. Because of the ease of measuring, the marginal fit is often emphasized above internal fit in the literature. For example, studies quantify the marginal fit of non-CADCAM restorations to range between 9 μm and 112 μm depending on the restoration type and material. This may be strongly influenced by the measurement method, restoration cementation, and the preparation design [1–5].

Although digital dentistry applications afford significant advantages to patients (such as crown preparation and delivery in one appointment and increased patient comfort with digital impressions), many clinicians are hesitant to adopt this new technology chairside. Previously, one main concern was the large marginal gap that was found on restorations milled by early generations of the CEREC systems [6, 7]. Practitioners may believe these outdated data still hold even with new digital CADCAM systems. Many studies agree that the fit of CADCAM restorations is comparable to non-CADCAM restorations, if not better [3–5, 8, 9]. According to recent meta-analyses [10, 11], single CADCAM restorations made by intraoral scanners (IOS) have similar marginal gaps as traditional elastomer impression methods.

Many factors influence the full digital approach, such as IOS brand, technology, scanning pattern [12–15], milling type and setting [16], preparation design and quality [17, 18], cement gap spacing [19], and software version [20]. There are chairside systems currently in use by practitioners but for which there exists very little published literature [16, 17, 21]. Therefore, there is a consistent need in the literature to evaluate the accuracy of dental scanning and milling systems with each successive product iteration.

Of important note is the limited availability of literature on digital scanning systems using true human tissue. Many studies compare scanners (and subsequently milled restorations) using typodont crown preparations [16, 19, 22]. However, because of the significant differences in material properties (including reflective index, hardness, and translucency), a typodont-based methodology likely diminishes the clinical significance of the findings [23, 24]. Additionally, a standalone typodont lack of neighboring teeth; therefore, the access of the proximal surfaces is not limited by the scanner head compared to the dentate arch [25].

Therefore, this study aimed to compare the accuracy of all-ceramic crowns fabricated by three IOS systems and their corresponding mills (CEREC Omnicam with MCXL mill; Planmeca Emerald with 40s and 30s mills). In order to mimic the clinical situation, a dentulous cadaver arch was used, and the scan was initiated at the contralateral side. The null hypothesis is that there is no difference in the marginal gap or the internal gap between crowns fabricated from CEREC Omnicam and MCXL and Planmeca Emerald and 40s and 30s systems in either detailed or standard mode.

## Methods

### Preparation of the samples

For comparison of scans on biological tissues, a human cadaver maxilla specimen was resected. The specimen contained hard and soft tissue and was preserved using methods previously described in detail [23]. The cadaver tissue used in this study was obtained from Anatomy Gifts Registry (AGR) is owned and operated by the Anatomic Gift Foundation, Inc. (Hanover, MD, U.S.). It is an independent, non-profit, anatomic donation organization that supports advancements in scientific research and medical education. All cadavers are donated to the AGR are obtained following written informed consent from the individual (1st person or willed authorization) or from a loved one (3rd person authorization). This study was deemed exempt from review by the Institutional Review Board for Human Research at the Medical University of South Carolina (Pro 77,251). According to this, no informed consent was necessary to use cadaver tissue.

An all-ceramic crown preparation by chamfer margin was performed on tooth 3. The preparation specifications of Rosensteil [26] and Shillingburg [27] were followed. A smooth, 1 mm modified shoulder finish line avoiding spikes and lips that follows the rise and fall of the gingiva was prepared using a diamond bur (8847KR-016, Brasseler, Savannah, GA, U.S.). The abutment was prepared rounded, and smoothed surfaces converged coronals by 10 degrees angle, with functional cusp bevel, 1.5 to 2 mm of occlusal reduction, 1 to 1.5 mm of axial reduction. Four full-arch scans were taken digitally using Planmeca Emerald (PE; Planmeca U.S., Roselle, IL) and four by CEREC Omnicam (CO; Dentsply Sirona, York, PA) IOS systems. All scans began contralaterally at tooth 15 and finished at tooth 3 [28]. Careful thought went into selecting an individual to scan with each scanner. For CEREC we had a CEREC trainer with 10 years of experience on CEREC, and for Emerald we had an Emerald trainer with also 10 years of experience. This was to remove intrinsic biases of having one person scan on both systems as invariably, users have a favorite system. By bringing in trainers for the systems to scan, we represent each scanner to the best ability. The scan patterns recommended by the manufacturers were applied. One master scan was obtained using the ATOS Capsule scanner (GOM, Braunschweig, Germany), which has demonstrated trueness of 3 μm and precision of 2 μm [29, 30]. Following scanning, tooth 3 was extracted using forceps extraction and stored in sterile saline away from light or heat.

### Restoration design

In order to standardize the size and shape of the milled restorations, a 3D-printed wax-up model of the maxilla specimen was created. The team used the master ATOS Capsule scan and digitally waxed tooth 3 to full contour using 3Shape laboratory software (3Shape Dental System 2017, 3Shape A/S, Copenhagen, Denmark). The waxed design was appended to the digital maxilla model, and the combined model was 3D-printed in plastic resin (Planmeca CREO C5 at 50 μm resolution, Planmeca Model Resin). This replica model was scanned using the Emerald and Omnicam scanners and used by the software as a template for the external surface of the crown designs. Therefore, across both systems, all crowns milled were standardized in external dimensions.

Crown designs were prepared on each IOS system’s respective software (Romexis, Planmeca, v5.2; CEREC software, Dentsply Sirona, v4.5). One digital crown design was designed for each scan of the maxilla.

### Milling

Three milling machines with three axes were used. The CEREC MCXL and Planmeca 40s have two spindles moving simultaneously, the Planmeca 30s has one spindle, and in both the MCXL and 30s, the milling blocks can rotate.

Each digital crown design was milled twice with each mill: one crown at the system’s slower setting mode (“detailed,” Planmeca; “fine,” CEREC) and the other at the system’s faster setting mode (“standard,” Planmeca; “fast,” CEREC). Planmill models use different burs (tools) for milling the internal surface of the restoration, depending on the setting. For the standard setting, an ellipsoidal bur with parallel axial walls and a flat end is used with a diameter of 1.6 mm (PlanMill Two Striper® Milling Diamond Burs Premier Dental Products, U.S.). In contrast, for a detailed setting, a conical bur is used with tapered axial walls. It has a rounded end and a smaller diameter at the tip (1.1 mm). CEREC uses the same tools for the fast and fine settings but producing more rough surfaces in case of a fast setting, according to the manufacturer. The intaglio was milled using the 12S Step Bur with a 1.3 mm diameter (Dentsply Sirona, Germany).

All crowns were milled of lithium disilicate ceramic blocks (IPS e.max CAD, size C14, color HT B2, Ivoclar Vivadent, Schaan, Lichtenstein) using new diamond burs and clean machines. Only one mill for each scan-mode-model (MCXL, 40S, 30S) was used for crown fabrication, for a total of 24 milled crowns.

Margin width was set at 250 μm (“margin ramp,” Planmeca; “margin thickness,” CEREC), and cement gap at 70 μm (“space thickness,” Planmeca; “spacer,” CEREC). All other parameters were accepted at default e.max parameters for each software. Sprue location was set slightly buccal of MB cusp and otherwise accepted at the software’s proposed location (i.e., coronal-apical position). Crowns were centered in milling block for standardization. Mill times were recorded from initiation of motor movement to visual confirmation on the device’s screen that milling was complete. All sprues were cut using a fine diamond bur to appropriate crown contour. Crowns were fired in an oven using the firing cycle recommended by the manufacturer (IPS e.max® CAD Speed crystallization, Ivoclar Vivadent, Programat CS) without prior application of gloss or stains.

### Micro-CT measurements of marginal and internal fit

**Fig. 1:**
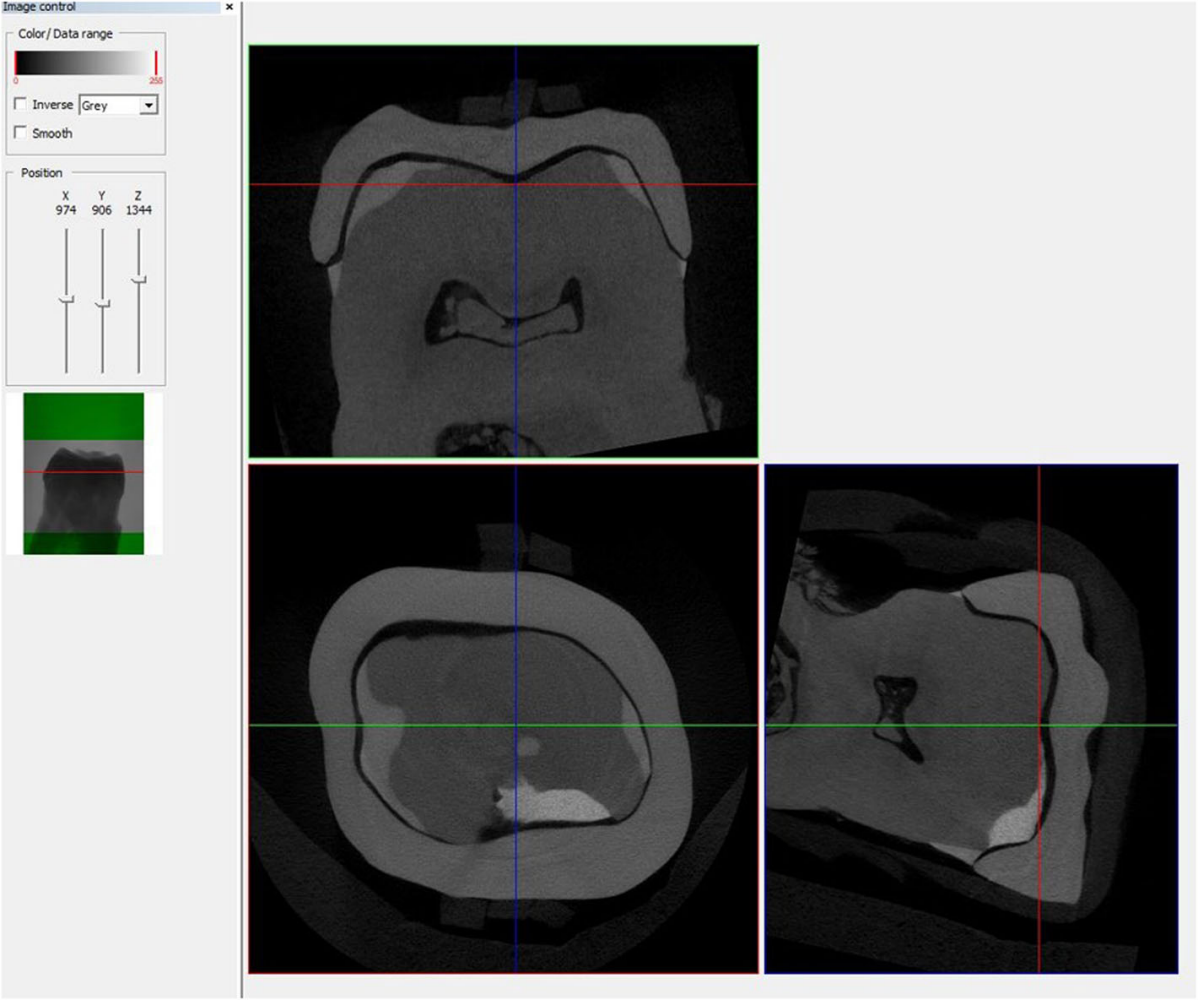
The section made in DataViewer in the identical position

Crowns were individually seated on the extracted tooth and tightly fixed by a rubber ring. Each specimen (crown and tooth) was scanned in a SkyScan 1172 micro-CT scanner (SkyScan, Aartselaar, Belgium). Images were acquired using 80 kV maximum accelerating voltage, 310 μA current, and 1 mm Al filter with a pixel size of 8.7 μm. The specimens were scanned at frames per rotation step of 0.7 (180°). After scanning, the images were reconstructed in software NRecon, which uses an FDK (Feldkamp–Davis–Kress) algorithm. DataViewer software (SkyScan, Aartselaar, Belgium, v1.5.2) was used to section each scan at the central region in the mesial-distal and buccal-lingual directions (Fig. 1). Because the same tooth was used in each scan, rotating the tooth to the same position allowed all scans to be sectioned in an identical position. The files were saved as .bmp format.

**Fig. 2:**
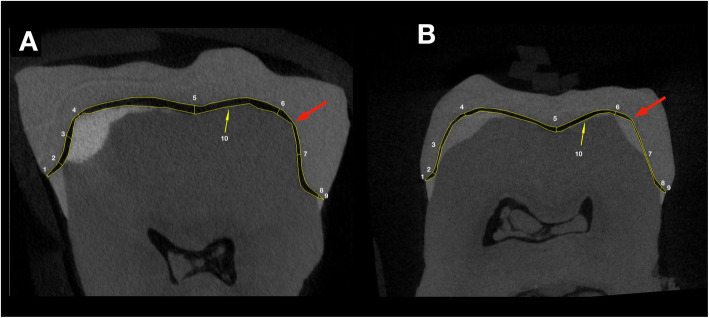
Sites of measurement on the mesio-distal section (**a**) and on the oro-vestibular section (**b**). The sites are numbered from mesial to distal and from oral to vestibular in the following manner: 1 - absolute marginal discrepancy (AMD), 2 - finish line (FL), 3 - axial wall (AW), 4 - axio-occlusal angle at cusp (CU), 5 - central of occlusal area (CO), and similarly 6, 7, 8, 9 on the other side of the tooth. The area selection between restoration and the tooth is indicated by N°10. A big red arrow indicates the possible point of the first contact of the restoration

The two sections were imported into ImageJ software (National Institute of Health, Bethesda, Maryland, USA, v1.52a) to make the gap measurements. The marginal and internal fit of the specimens were assessed as recommended by Holmes et al. [31] at various points: absolute marginal discrepancy (AMD), finish line (FL), axial wall (AW), axio-occlusal angle at cusp (CU), and central of occlusal area (CO). The points were measured buccally, lingually, mesially, and distally using both scan sections (Fig. 2). The perimeter between the restoration and tooth was also measured to calculate an average full gap between the two surfaces (Fig. 2). The measurements were repeated five times by five observers without knowing the group specification of the specimen (blinded analysis). They were not involved in either in the fabrication process (scanning or milling) or in the microCT measurement. Similarly, the microCT measurement was done by two investigators not involved in other processes, and the sectioning of the microCT scan in DataViewer was done by a single observer who was not involved in other processes either.

### Statistical analysis

The 100 data points per specimen were exported to MS Excel for organization and then exported into SPSS 25 (IBM SPSS Statistics for Windows, Version 24.0. IBM Corp., Armonk, NY., U.S.) for further statistical analysis. Gap data were analyzed with the generalized linear mixed-model approach with gamma distribution and log-link function using restricted maximum likelihood estimation. In the complex design, the site, the mill model, and the mill mode (standard/fast vs. detailed/fine) were the main fixed factors, with their interactions integrated into the model. The section was included in a second design to test the difference in AMD between sections. The *p* values were adjusted using the Bonferroni method for pair-wise comparison with an alpha value set at 0.05. Data in the text and figures are presented as estimated marginal mean ± standard error.

The inter-rater reliability was evaluated in two ways. The relative reliability was assessed by the intraclass correlation coefficient (ICC) [32]. The absolute agreement form of ICC was utilized as both the trueness and precision of the measurements were considered [33, 34]. ICC values of < 0.40, 0.40–0.75, and > 0.75 were considered as poor, fair-to-good, and excellent agreement, respectively [35]. The reliability was also assessed by the calculation of the within-subject coefficient of variation [36]. It measures reproducibility by determining the degree of closeness of repeated measurements taken on the same subject by the different observers under the same condition. It gives estimates for the magnitude of the error during distance measurement on the section made by the observer. The coefficient of variation (CV) was calculated from the variance component (VC) of the log-transformed variable using the following formula $CV=100\times \sqrt{VC}$ [37], with a confidence interval of 95%. CV values of ≤10%, 10–25%, and ≥ 25% were considered as good, moderate, and poor reliability, respectively [35].

## Results

### Inter-rater reliability and variance components of the measurements

The ICC was 0.88, which suggests excellent agreement between the observers. The reliability of averaged measurement increased to 0.97. The CV was 21% [20.8-22.1%] for the observer, 9% [2.8-30.5%] for the section, and 9% [3.2-25.2%] for the scan (no differences between Omnicam and Emerald).

### Full gap analysis

**Fig. 3:**
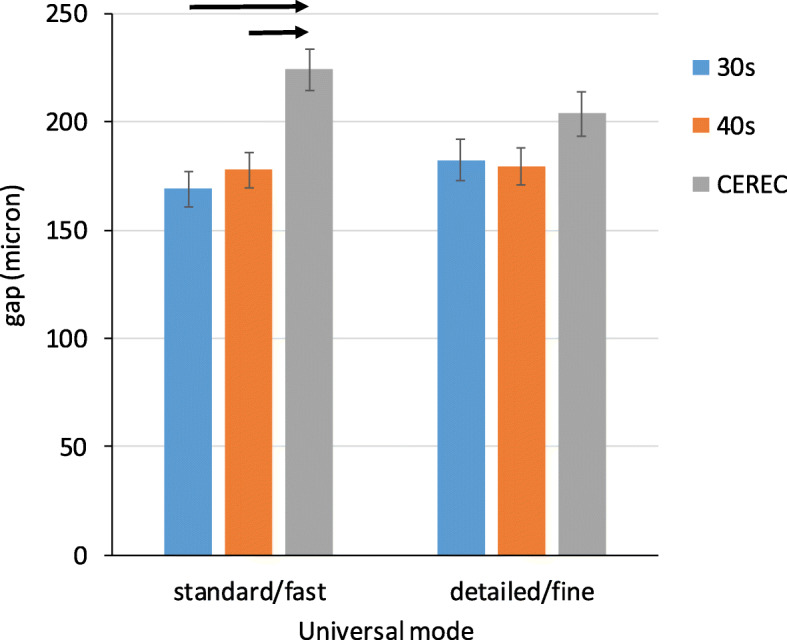
The full gap between crowns and tooth abutment in microns. Significant differences between models are denoted by black arrows, *p* < 0.001

The mill mode did not influence the size of the full gap in either model (*p* = 0.926, Table 1). Significantly higher full gap value was observed with CEREC than with Planmeca models in standard/fast mode (Fig. 3), but not in detailed/fine mode (30s vs. CEREC *p* = 0.239, 40s vs. CEREC *p* = 0.206). No differences were observed between 30s and 40s models in either mode (standard *p* = 0.168, detailed *p* = 0.713).

**Table 1:** Gap measured between crowns and abutment

		universal mode			
		standard/fast		detailed/fine	
site	model	Mean	SE	Mean	SE
full	30s	169	8.1	182	9.3
	40s	178	8.5	180	8.6
	CEREC	224	9.6	204	9.9
AMD	30s	66	8.5	76	9.9
	40s	74	9.6	83	10.7
	CEREC	133	14.6	130	17.0
FL	30s	180	23.4	197	25.7
	40s	194	25.3	180	23.5
	CEREC	125	13.7	116	15.1
AW	30s	112	14.6	134	17.5
	40s	112	14.6	142	18.5
	CEREC	120	13.1	120	15.6
CU	30s	125	16.3	169	22.0
	40s	152	19.8	146	19.0
	CEREC	236	25.8	210	27.5
CO	30s	248	33.7	302	41.0
	40s	271	36.8	259	35.2
	CEREC	391	44.5	320	43.7

### Gap measurement at various sites

The three-way interaction of mode*site*model was not significant (*p* = 0.949), as well as the two-way interactions of mode*model (*p* = 0.154) and mode*site (*p* = 0.685). The main effect of mode was not significant (*p* = 0.423). A significant effect was observed for the main effect of the site (*p* < 0.001) and the interaction of model*site (*p* < 0.001). Therefore, further pair-wise comparisons were made between sites and between separate models at various sites.

**Fig. 4:**
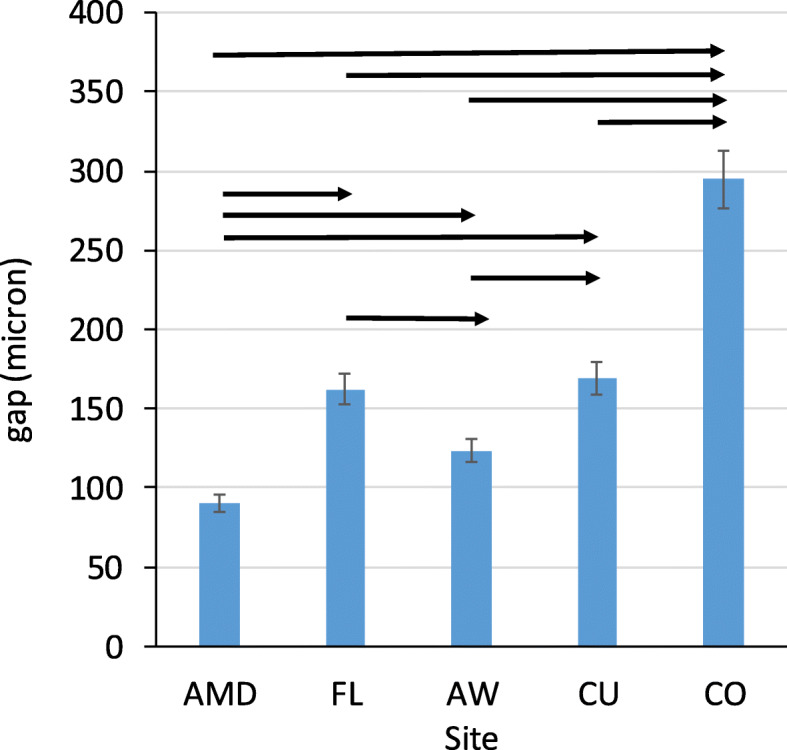
The gap between crowns and tooth abutment in microns at various sites. Significant differences between sites are denoted by black arrows, *p* < 0.001

The gap was significantly higher at CO than at all other sites (*p* < 0.001 for each pair, Fig. 4). No difference was observed between CU and FL (*p* = 0.468). Both were significantly higher than AW and AMD (*p* < 0.001 for each pair). The next lower values were at AW, but it was significantly higher than AMD (*p* < 0.001). The order of the gap size was as follows: CO > CU=FL > AW>AMD.

**Fig. 5:**
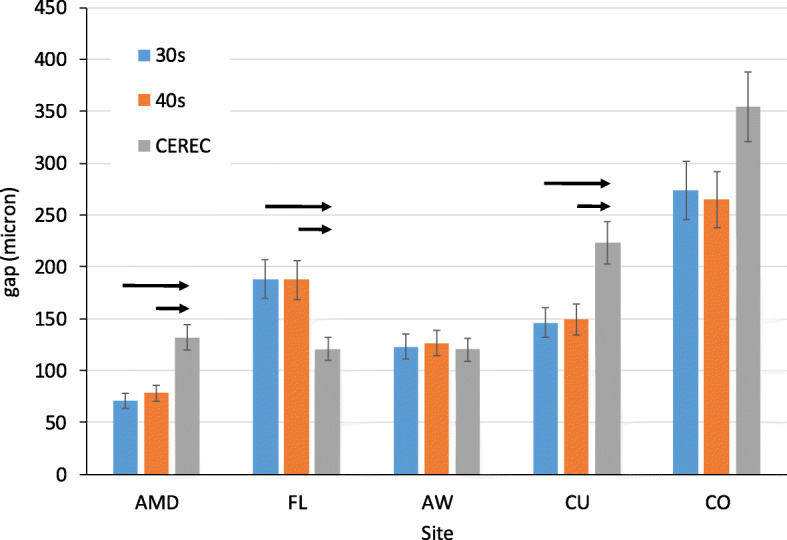
The gap between crowns and tooth abutment by site and mill system in microns. Significant differences between models by the site are denoted by black arrows, *p* < 0.01

No differences were observed between the 30s and 40s at any sites (Fig. 5). No differences were observed between Planmeca and CEREC models at the axial wall (AW) and central occlusal fossa (CO). CEREC had a higher value at absolute marginal discrepancy (AMD) and cusps (CU) than Planmeca models. Planmeca models had a higher gap at the finish line (FL) than CEREC.

### Differences in AMD between sections

In the three-way factor analysis (model*mode*section), no interactions were found to be significant, and only the main effect of the section was significant. Thus, regardless of mode and models, the mean AMD on the coronal section (133 ± 21 μm, oral and vestibular measurements) was significantly higher than on the sagittal section (55 ± 8.6 μm, mesial and distal measurements, *p* < 0.001).

### Time of milling

There was no difference between actual and predicted times with the two Planmeca models (30s: 25.2 ± 2.5 min vs. 25.2 ± 2.5 min; 40s: 13.3 ± 1.4 min vs. 13.3 ± 1.4 min). In the case of CEREC, the actual time was significantly higher than the predicted time (13.4 ± 1.2 min vs. 10.0 ± 0.9 min, *p* < 0.001). The 30s had significantly higher actual and predicted time compared to all other models (*p* < 0.001). No significant difference was observed in either actual or predicted time between the 40S and CEREC models. In the standard mode, the average milling time was 7.2 min less than in the detailed mode (13.3 ± 0.97 min vs. 21.8 ± 1.87 min, *p* < 0.001) regardless of the model and whether the predicted or actual milling time was considered.

## Discussion

McLean and Fraunhofer, in their landmark in vivo study, quantify a marginal gap of 120 μm to be clinically acceptable [1]. However, experienced dentists accepted marginal gaps as great as 455 μm while rejecting marginal gaps as small as 117 μm [38]. Interestingly, in vitro studies failed to demonstrate a correlation between the marginal gap and leakage [39], even when the gap ranged between 0 and 831 μm [40]. However, a strong correlation can be found between gingivitis and marginal discrepancy ranging from 5 to 430 μm [41], suggesting that measuring the AMD has a higher relevance than the marginal gap. Restoration longevity is not compromised with marginal gaps up to 120 μm for conventional restorations luted by polycarboxylate cement [42] and the marginal gaps above 200 μm for CADCAM or heat-pressed all-ceramic crowns cemented by adhesive technique [43]. Notwithstanding the lack of direct evidence for a relationship between the marginal gap and restoration longevity, the CADCAM restorations had good clinical success [43–45]. Therefore, some authors [2, 46] question the necessity of the cement film thickness is less than 120 μm. Similarly, it was found that a significant proportion of marginal gaps were above 150 μm in the case of the gold cast [47] despite the well-known high clinical success rate of this restoration [48, 49].

Studies comparing the marginal gap of CADCAM ceramic restorations made from digital and conventional impressions [10, 11] showed that the mean marginal gap of the digital workflow is in a range between 18 and 128 μm. Within the included studies, there was a significant variety in preparation design, specimen (an extracted tooth or typodont), milling unit, evaluation method (microCT, silicone replica technique, etc.), and marginal fit measurement (AMD vs. marginal gap). The preparation design (rounded vs. chamfer) does not affect the gap size, according to a meta-analysis [11]. In this study, the AMD measured 71-78 μm for Planmeca models and 132 μm for CEREC, which appears higher than the range of studies included in the meta-analysis. However, in most studies [5, 16, 17, 50], the marginal gap was measured, defined by Holmes to be the closest measurement from the internal surface of the restoration to the wall of the preparation at the margin [31]. Similar to this study, others [19, 22, 51] measured the absolute marginal discrepancy, which is the distance between the preparation margin and restoration margin. AMD is the summation of the horizontal and vertical marginal discrepancy vectors. This is equivalent to the addition of the marginal gap and overextended margin vectors. This means that AMD is higher than or in an ideal condition perhaps equal to the marginal gap. In studies measuring both parameters, the AMD was found to be 30-118% greater than the marginal gap [52–54]. Literature regarding marginal fit with chairside systems is available for CEREC, but most of them scanned typodont specimens. The mean marginal gap ranged from 94 to 145 μm [16, 17]. These results are comparable to this study’s CEREC data, considering the difference between the marginal gap and AMD. However, other studies measured AMD with CEREC systems and obtained lower values, between 53 and 94 μm [19, 22, 53]. The lower values of these studies may come from ideal in vitro conditions obtained by scanning a single typodont tooth instead of a full dentate arch dentate. In an in vivo study [55], the mean marginal discrepancy was 149 μm. This is similar to CEREC results presented here, suggesting that the scanning condition may have a contribution to the error besides the effect of the milling machine and restoration material.

A recent study [56] found that there is a significant difference between the five methods of measuring the marginal fit, and the values obtained by microCT are located in the middle of the five techniques. The results of microCT studies are very similar to those presented here. The mean AMD was 151-161 μm with the Omnicam and CEREC inLab milling unit [57] and 145-412 μm after conventional impression [52]. The microCT with high resolution increases the contrast of the margin line of the specimens, which could improve the measurement. In this study, an extremely high resolution (8.7 μm) was used, higher than previous studies with 10-19 μm resolution [52, 57–59]. The high resolution could contribute to the significant findings in our study despite the low sample number. Groten et al. [60] determined the optimal amount of measurement sites for getting a consistent AMD value in the case of microscopic observation. Their recommendation, making 50 measurements of each sample, became a standard for evaluating AMD by using a microscope [5]. The microscopic evaluation inherently involves projection error due to the frontal view contrary to the sectional view used in microCT. Furthermore, the standard selection of measurement points around the multiple replicas is challenging. In our study, the same abutment was used for each crown, and standardized positions were used for sectioning. The common problem in both methods is that the selection of the starting point and endpoint of the distance measurements is somewhat subjective. In our study, five observers made repetitive measurements in the same section to overcome this type of error. The magnitude of the variation between observers indicated a significant contribution of the distance measurement to the overall error. The involvement of multiple observers allows controlling this error in the statistical model. The whole coefficient of variation in AMD measurement was between 22 and 26% in our study, which was similar to the CV in recent microCT studies [52, 57–59, 61]. In conclusion, these results highlight the importance of involving multiple observers in the analyses and indicate the high accuracy of the microCT method.

Manual adjustment or discarding of restorations after visual inspection could greatly decrease the marginal gap and AMD [57], but this technique was not applied in this study contrary to other studies [4, 8, 55, 57, 62, 63].

Overall, the greatest difference, which distinguishes this experimental design from all others, is the scan of a dentate human maxilla compared to a standalone typodont made of plastic. Typodont abutments are often more easily accessible, especially at the approximal surfaces, for scanning than abutments in a dentate arch [25]. This phenomenon may contribute to the in vivo-like AMD values, especially with the CEREC system. Interestingly, the AMD was consistently smaller in the mesiodistal than in the buccolingual section. The variance component analysis revealed that the variance between scans had little contribution to the overall error. Furthermore, no difference in this variance component was observed between Omnicam and Emerald. Therefore, the difference between sections and systems may relate to some milling discrepancy instead of the scanning differences, but the answer to this question requires further studies.

The mean full internal gap was between 169 and 182 μm for Planmeca systems and higher for CEREC at 204-224 μm. The lowest value in the internal surface was at the axial walls and the highest at the occlusal walls. This is similar to other studies, regardless of conventional [4, 64, 65], or digital impression was used [16, 59, 66]. Milling inaccuracies might predominantly occur on the occlusal surface of the CADCAM restorations because of preparation errors and irregularities [16, 59] and digital techniques that may round sharp edges [4]. Axial walls are milled with the side of the instrument bur. In contrast, the inner occlusal surface is milled with the tip of the bur, perhaps exaggerating dimensional discrepancies between instrument size and inner geometry of the restoration.

Mostly, adjustment of the cement spacer could explain the marginal gap being lower than the axial and occlusal gaps. In both conventional casting and CADCAM techniques, no space is allocated at the marginal ramp area, and tens to hundreds of microns of space are created at axial and occlusal walls. With the conventional casting technique, the marginal gap was the largest when no spacer was used and dramatically decreased when multiple spacer layers (up to 70 μm) were used [67]. Many studies demonstrated that too thin of a die spacer might inversely increase the gap, likely due to the lack of cement release [68–71]. Similarly, in the case of the CEREC system, increasing the spacer setting from 10 μm to 100 μm resulted in a lower AMD [19, 20, 72]. One study showed 200 μm spacer settings resulted in the lowest marginal gap in e.max crowns made by E4D mill system [73]. In this study, a cement space of 70 μm and a margin ramp of 250 μm was set for both systems. CEREC manual (Software version 4.6.x) recommends 120 μm for the spacer, which is higher than applied in this study. Planmeca FIT CADCAM system’s manual recommends 100 μm spacer thickness [18]. The spacer setting could compensate for the inaccuracies of the fabrication workflow to minimize the marginal gap. Therefore, a lower spacer setting than factory recommendation may inversely increase the gap, possibly explaining the margin values found to be higher with CEREC than Planmeca.

The internal gap and the cement thickness may influence the retention and fracture resistance of restoration. A very early study [74] found only a very weak correlation between retention of the restoration and zinc phosphate cement thickness in a range of 20-140 μm. The bond strength of porcelain cemented by resin was not changed by increasing cement thickness from 50 μm to 200 μm [75]. In another study, increasing the resin cement thickness from 50 μm to 100 μm decreased the shear bond strength of the disks by 20-43% [76]. With lithium disilicate ceramic crowns and resin cement, the pulling test resulted in a fracture of the restoration before any adhesive debonding 90% of the time [77]. It suggests that dual cement is highly retentive, and thus the thickness of the cement within a certain range may be less important. When increasing the cement thickness of resin from 50 to 500 μm, the fracture resistance of the feldspathic crown decreased significantly [63, 78]. No significant effect was seen on fracture resistance of IPS e.max CAD milled by CEREC system with cement thickness ranging from 30 to 150 μm [79]. In a range of 26-297 μm, only a slight decrease in fracture strength was observed in glass-ceramic Tabs [80]. According to these data, the internal gap settings of both systems in this study were within an acceptable range.

A difference in accuracy was observed between CEREC and Planmeca systems but not between Planmeca models. Planmill 30s has one 3-axis spindle, and the object can rotate, whereas the 40s and MCXL models have two spindles moving simultaneously in 3 axes. Studies show that the number of axes does not necessarily improve the accuracy of the restoration [54, 59, 81, 82], which is confirmed by the results presented here. The Planmill 30s required more time for milling, likely due to its one spindle. The gap after the whole workflow in this study was likely more dependent on factors described above, such as scanning accuracy of natural hard and soft tissue, finish line distinctness [83], and mill settings. In one study, a significant difference was found between two intraoral scanners in the marginal gap of lithium disilicate crowns despite using the same milling unit, Planmill 40 [21]. They also found a higher gap at buccal and lingual than mesial and distal sites for Planscan, similarly as in this study. As errors could compound through the workflow [84], the importance of researching every step in the workflow must be emphasized.

No difference was observed in gaps between modes of milling (detailed/fine vs. standard/fast). One study demonstrated a significant difference in the marginal gap between detailed and standard mode of Planmill 40 [21], but as discussed previously, a typodont model was used. Research of different CAM strategies (e.g., different bur sizes) of CEREC chairside system shows no statistical differences in the measured gap [16]. E.max crowns fabricated by E4D system had a mean vertical marginal gap of 38 μm with ideal preparations and 90 μm with poor preparations [18]. This range is similar to the results of this study (85-96 μm for Planmeca models) when taking into account the estimated differences between AMD and marginal gap. It is possible that the lack of difference in accuracy between modes in this study may be a result of continuous software development and the ideal tooth preparation, which could avoid under- or over-milling the internal surface by a larger bur. In this experiment, all crowns were fabricated on the same tooth prepared by an experienced dentist. As the milling took longer in detailed/fine mode, it may be of little benefit to using this mode with a well-prepared abutment.

No difference was found between predicted and actual times in either Planmeca models, suggesting a well-developed simulation software. However, for CEREC, the times were underestimated by an average of 3 min. The researcher needs to note that software for both systems is continuously developed and upgraded, which may influence the speed, accuracy, and estimated mill time of restorations [20].

## Conclusions

In this study, it was attempted to simulate the workflow of manufacturing a chairside ceramic restoration from the scanning to try in. The mean marginal discrepancy was at a clinically acceptable level for both systems, though Planmeca models had significantly better performance. The cheaper and simpler model, the Planmill 30s, had the same performance as the Planmill 40s, though it had slower processing times. Interestingly, the time-consuming detailed milling setting had no significant improvement to the fit of full-ceramic crowns. It is necessary to further investigate the entire chairside process with different teeth and preparations to scrutinize the benefit of detailed/fine mode.

## Data Availability

The datasets used and/or analyzed during the current study are available from the corresponding author upon reasonable request.

## Abbreviations

CADCAM: Computer-aided design/computer-aided manufacturing
IOS: Intraoral scanner
AMD: Absolute marginal discrepancy
FL: Finish line
AW: Axial wall
CU: Axio-occlusal angle at the cusp
CO: Central of occlusal area
FULL: Full internal fit
